# Structure of Biocompatible Coatings Produced from Hydroxyapatite Nanoparticles by Detonation Spraying

**DOI:** 10.1186/s11671-015-1160-4

**Published:** 2015-12-01

**Authors:** Valentyna Nosenko, Nataliia Strutynska, Igor Vorona, Igor Zatovsky, Volodymyr Dzhagan, Sergiy Lemishko, Matthias Epple, Oleg Prymak, Nikolai Baran, Stanislav Ishchenko, Nikolai Slobodyanik, Yuriy Prylutskyy, Nickolai Klyui, Volodymyr Temchenko

**Affiliations:** V. Lashkaryov Institute of Semiconductor Physics, National Academy of Sciences of Ukraine, 45, Pr. Nauky, Kyiv, 03028 Ukraine; Taras Shevchenko National University of Kyiv, Volodymyrska Str., 64/13, 01601 Kyiv, Ukraine; National Technical University of Ukraine “KPI”, 03056 Kyiv, Ukraine; Inorganic Chemistry and Center for Nanointegration Duisburg-Essen (CeNIDE), University of Duisburg-Essen, Universitaetsstrasse, 5-7, 45117 Essen, Germany

**Keywords:** Calcium phosphate, Hydroxyapatite coating, EPR, Raman, XRD, SEM, Detonation spraying

## Abstract

Detonation-produced hydroxyapatite coatings were studied by scanning electron microscopy (SEM), X-ray powder diffraction (XRD), Raman spectroscopy, and electron paramagnetic resonance (EPR) spectroscopy. The source material for detonation spraying was a B-type carbonated hydroxyapatite powder. The coatings consisted of tetracalcium phosphate and apatite. The ratio depended slightly on the degree of crystallinity of the initial powder and processing parameters of the coating preparation. The tetracalcium phosphate phase was homogeneous; the apatite phase contained defects localized on the sixfold axis and consisted of hydroxyapatite and oxyapatite. Technological factors contributing to the transformation of hydroxyapatite powder structure during coating formation by detonation spraying are discussed.

## Background

Titanium is the most widely used implant material in orthopedic surgery and stomatology due to its durability under high load, as well as the applicability in complex mechanical systems, such as knee and elbow joints. However, there are some challenges in titanium implant applications, such as possible degradation under the corrosive action of biological tissues in combination with continuous and/or cyclic loads, harmful action of electrochemical products of corrosion, and metal sensitivity of the human body [1, 2]. To overcome these disadvantages, orthopedic and stomatological titanium implants are coated with ceramics which provides good biocompatibility with living tissues. The materials commonly used for producing such ceramics are various calcium phosphates, especially hydroxyapatite (HAP), Ca_10_(PO_4_)_6_(OH)_2_ [3–6]. Titanium-based implants coated with HAP facilitate quick bone adaptation and allow to firmly fasten the implant to the bone and to significantly reduce the time of healing. The good biocompatibility of such coatings is caused by the fact that HAP is the mineral phase of bone (~60 %) and teeth (~85 %) [3, 7]. To further improve the biocompatibility, the coating properties must be as close to natural tissue characteristics as possible. Taking into account that the biological HAP contains at least 4–6 wt % of carbonate impurity, the aim of the present study was to obtain a carbonate-containing HAP (CO_3_^2−^-HAP) coating.

Among various methods to obtain HAP coatings, we have chosen the detonation spraying method [8]. Its advantages are the ability to coat surfaces of arbitrary shapes and/or large-scale surfaces, and the method provides a good adhesion to the substrate. However, this coating technique can change the phase composition, structure, and other properties of the initial material. The changes can occur during either explosion (temperature-induced changes) or deposition on the titanium substrate (impact-induced changes). We report on the characterization of these coatings and analyze how the properties of the source material and technological parameters influence on the properties of the coatings. This knowledge is very important for control of biological performances of the coatings used as implants.

## Methods

### Sample Preparation

The source material for coating fabrication was carbonate-containing hydroxyapatite. It was synthesized by wet precipitation method using Ca(NO_3_)_2_ · 4 H_2_O, Na_2_CO_3_, and Na_3_PO_4_ · 12 H_2_O as the initial components. The solution with a mixture of sodium carbonate and sodium phosphate was pumped into a reactor containing a calcium nitrate solution (0.1 M). The molar ratio of Ca/P was 1.67:1, corresponding to stoichiometric hydroxyapatite. The molar ratio of CO_3_^2−^/PO_4_^3−^ was 1:1. The obtained amorphous precipitates were filtered and washed several times with water to eliminate any residual alkali ions. The samples were prepared at different conditions at 25 or 80 °C (hereafter denoted as powder P1 and powder P2, respectively). Then, the samples were dried at 80 °С. The well-ground powders were used to coat Ti plates.

A detonation spraying setup was used for deposition. The coating was generated by igniting an explosive mixture of oxygen and combustible gas (propane-butane) in the explosion chamber. The wave propagated along the gun tube and caught up a portion of the HAP powder injected into the gun. Particles of the material were accelerated up to 5 M of speed (M is the Mach number ~340 m/s) and bombarded the substrate, forming a continuous coating due to physical and chemical interactions with the substrate material. The programmable displacement of the detonation gun or the substrate provided coating of a large area or multi-surface substrates. The coating of plates was done at two different distances from the gun to the titanium plate. Table 1 gives a short description of the studied samples.

**Table 1:** Description of the studied samples

Sample	Description
P1 (powder)	Synthesis at 25 °С
P2 (powder)	Synthesis at 80 °С
T1 (coating)	Spraying of P2; distance to the Ti plate—150 mm
T2 (coating)	Spraying of P2; distance to the Ti plate—200 mm
T3 (coating)	Spraying of P1; distance to the Ti plate—150 mm
T4 (coating)	Spraying of P1; distance to the Ti plate—200 mm

### Methods

X-ray powder diffraction (XRD) was used to analyze the phase composition of the samples. Diffractometers type Shimadzu XRD-6000 and Bruker D8 ADVANCE with Cu K_α_ radiation were used. Data were collected over the 2*θ* range 5.0°–90.0°, with the steps 0.02 and 0.01°. Identification of the phases was achieved by comparing the diffraction patterns of the samples with ICDD standards. Scanning electron microscopy (SEM) (FEI Quanta 400 ESEM instrument in a high vacuum after sputtering with Au:Pd) was applied to observe the morphological changes of the coatings. Raman spectroscopy was used to identify the chemical composition and molecular structure. Raman spectra of the initial powders and deposited coatings were investigated at room temperature using a DFS-24 double monochromator and a photon-counting system for recording. The radiation of a CW argon ion laser (514.5 nm, 50 mW) was used for excitation. Electron paramagnetic resonance (EPR) spectroscopy was applied to analyze structural changes on the atomic level. EPR spectra were measured with a Radiopan X-band EPR spectrometer (~9.5 GHz). EPR signals were recorded at 2 mW microwave power using 100 kHz modulation of magnetic field with 0.05 mT amplitude (spectra of the samples were recorded together with the spectrum of MgO:Cr^3+^ reference sample (*g* = 1.9800) that allowed to compare the intensities of the EPR spectra of different samples). The estimated accuracy of the g-factor determined for the EPR lines observed was ±2 · 10^−4^.

## Results and Discussion

### SEM and XRD Studies

**Fig. 1:**
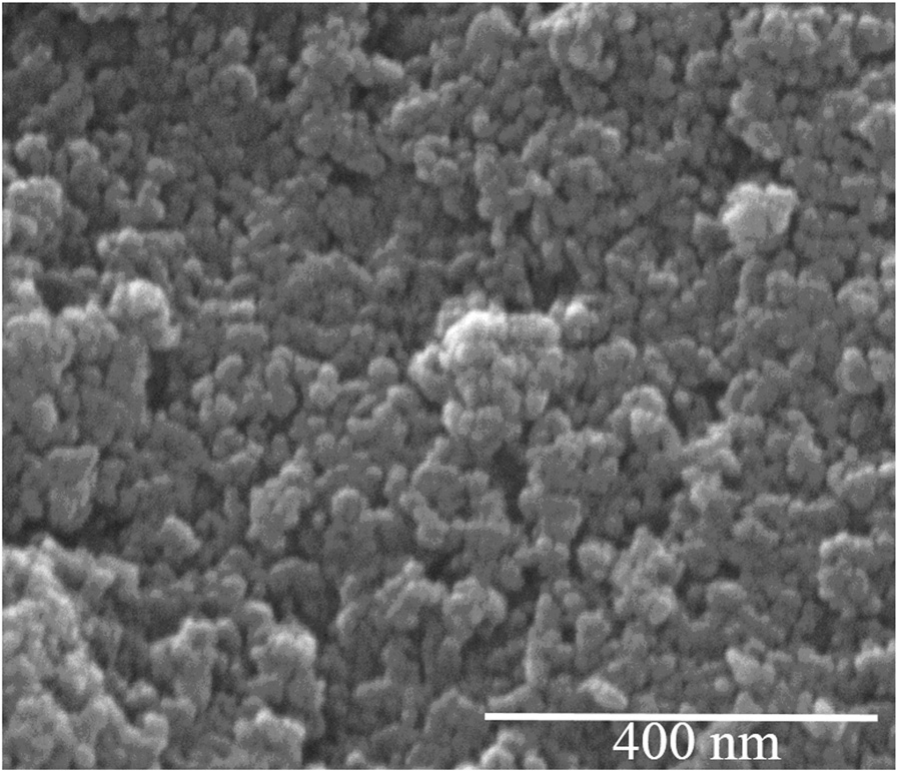
Scanning electron micrograph of sample P1. P1 powder prepared at 25 °С

The morphology of the initial powders was studied by scanning electron microscopy. For both powders (P1 and P2), SEM showed similar structures: the powders consist of irregular spherical particles with the sizes about 20–30 nm. The SEM image of P1 sample is shown as an example (Fig. 1).

**Fig. 2:**
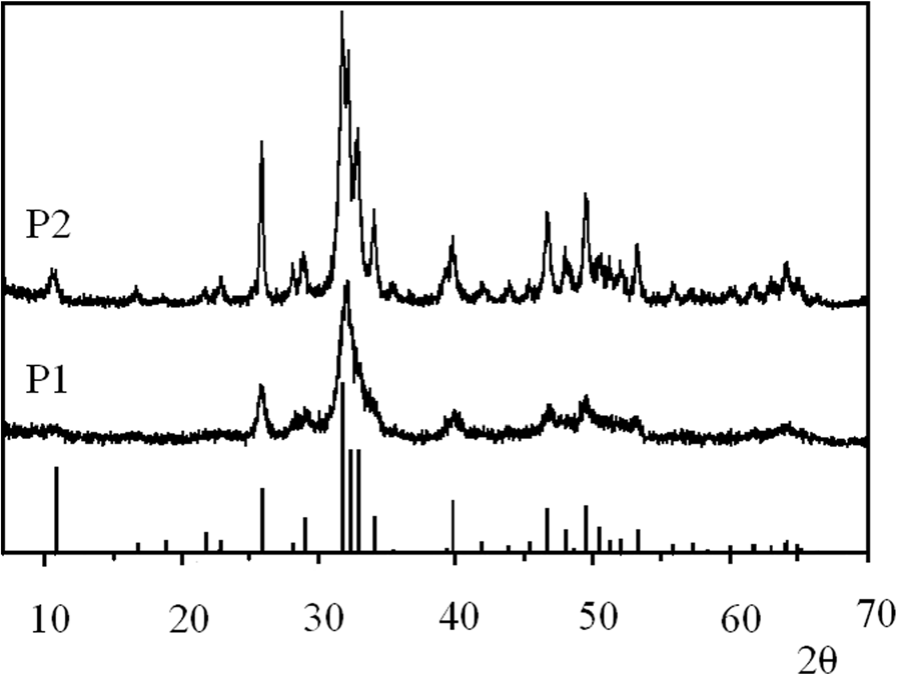
X-ray diffraction patterns of carbonated apatite powders P1 and P2. The calculated positions of main HAP reflections are shown by *vertical lines* (from ICDD (#00-089-6495))

The X-ray diffraction patterns of the powders P1 and P2 are shown in Fig. 2, together with the reported peak positions for HAP (ICDD pattern #00-089-6495). They corresponded to a crystalline HAP phase with a hexagonal unit cell and lattice parameters *a* = 9.432 Å and *c* = 6.881 Å. It is seen that the XRD patterns of P1 and P2 powders are typical patterns of an HAP structure (Fig. 2). All reflexes are significantly broadened which is due to the small particle size (Fig. 1) and to the poor crystallinity of the HAP. The XRD patterns of P1 and P2 samples are similar; however, for P1 all reflexes are slightly broadened, suggesting that crystallinity of P2 powder is better than that of P1.

**Fig. 3:**
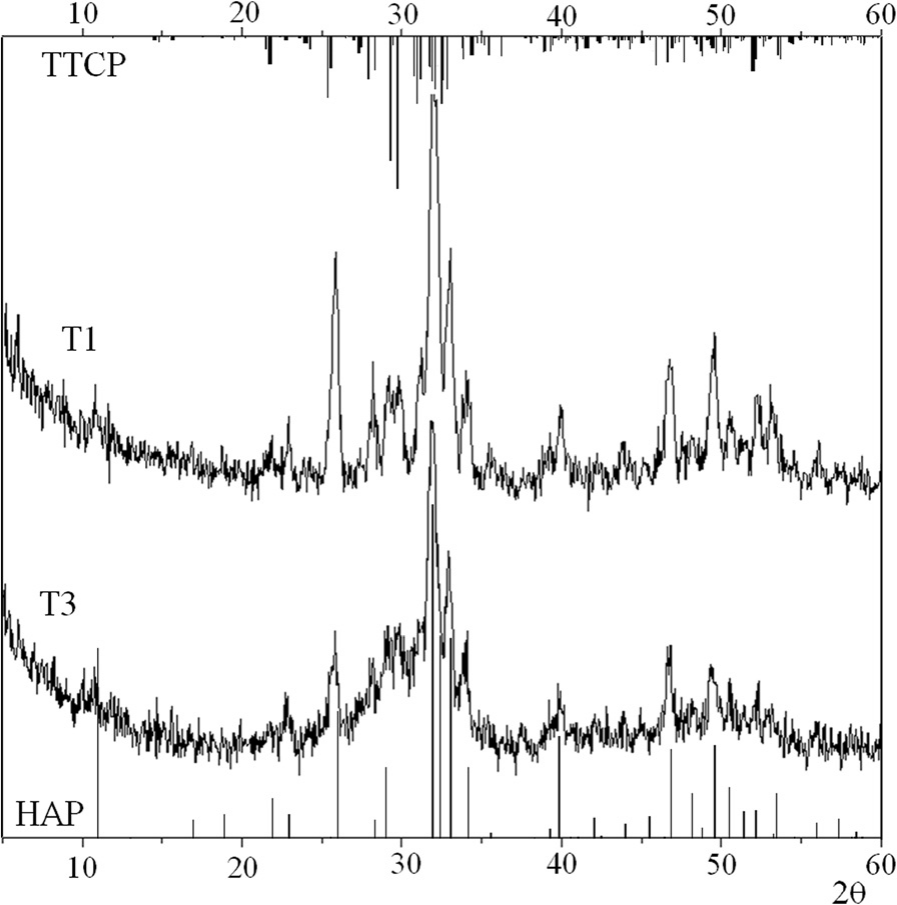
X-ray diffraction patterns of the samples T1 and T3.The calculated positions of HAP (#00-089-6495; *bottom*) and TTCP (#00-070-1379; *top*) reflections are shown by *vertical lines*

**Fig. 4:**
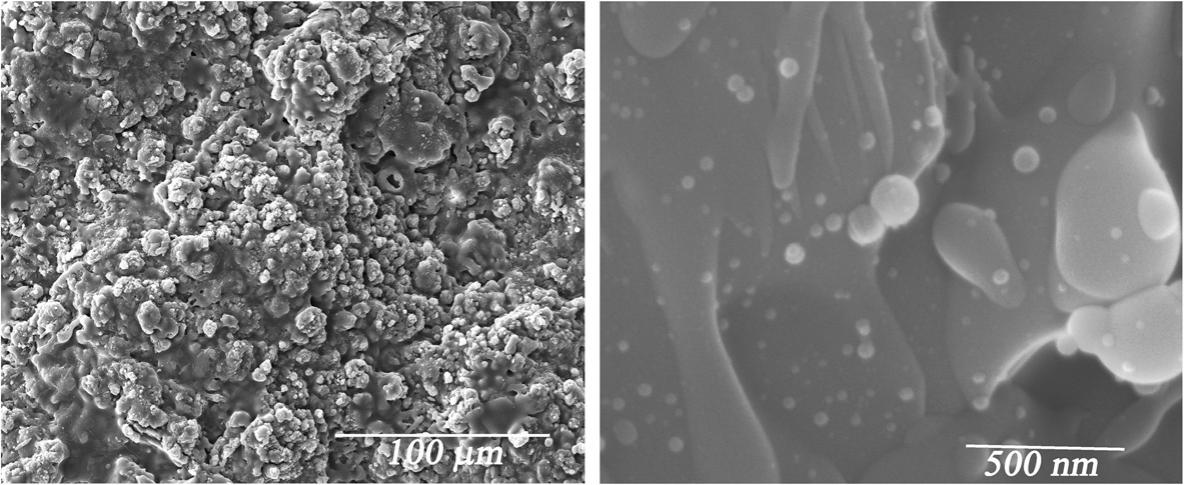
Scanning electron micrograph of the coating T1

Figure 3 shows the X-ray diffraction patterns of carbonated apatite coatings on Ti plates (T1 and T3 samples). The narrower reflections as compared to the XRD patterns in Fig. 2 indicate the increase of crystallinity. XRD patterns of all coatings show both the expected HAP reflexes and some additional peaks that are most evidently observed in the range 27°–31°. These peaks can be attributed to tetracalcium phosphate (TTCP) phase, Ca_4_(PO_4_)_2_O; the positions of the corresponding reflections are shown in the upper part of Fig. 3. The example of morphology of an obtained coating is demonstrated in Fig. 4.

Figure 3 shows that the T1 spectrum in comparison with T3 demonstrates narrower reflexes and better resolution. This indicates that the T1 coating has a higher crystallinity.

Lattice parameters for both phases of all coatings were calculated from the XRD data. The TTCP lattice parameters were the same for all samples (monoclinic system, space group *P*2_1_, *а* = 7.023 Å, *b* = 11.986 Å, and *c* = 9.473 Å, *β* = 90.9°), whereas small changes were observed for the HAP phase (see Table 2). The parameter *а* for all coatings was considerably smaller than the value of *a* = 9.432 Å of B-type HAP. Since the value *а* for the stoichiometric (without carbonate) HAP is 9.4176 Å [7], it can be assumed that, first of all, the variation of *а* is related to the partial escape of water and carbonate during the process of coating deposition. Furthermore, as it is seen from Table 2, the calculated values of the lattice parameter *a* for all samples are slightly smaller than *a* = 9.4176 Å; this decrease can be caused by the formation of some structural defects such as vacancies. Note that a value of *a* smaller than 9.4 Å is often observed in B-type carbonate-containing apatite annealed at different temperatures (see, for example, [9]). Previously in [19], it was shown that the high-temperature annealing (above 700 °С) of carbonate-containing HAP causes partial escape of carbonate and structural-bound water. As a result, HAP transformed partially into TTCP. During the detonation spraying, this process apparently takes place in the surface layers of the powder grains, which are subjected to maximum temperature action.

**Table 2:** The calculated cell parameters of HAP phase in the coatings

Sample	Lattice parameters	
	*a* (Å)	*c* (Å)
T1	9.405(2)	6.902(2)
T2	9.400(3)	6.903(2)
T3	9.413(4)	6.905(4)
T4	9.406(3)	6.904(3)

### Raman Spectroscopy

The values of the frequencies of PO_4_^3−^ in water obtained from Raman scattering measurements are *ν*_1_ = 936.6 cm^−1^, *ν*_2_ = 415 cm^−1^, *ν*_3_ = 1010 cm^−1^, and *ν*_4_ = 558 cm^−1^ [10]. In the case when the PO_4_^3−^ tetrahedron is a part of symmetrical lattice HAP and/or TTCP, the crystal field induces distortions in PO_4_^3−^ tetrahedron which change the intra-tetrahedral bond lengths and angles, and as a result, the normal modes of PO_4_^3−^ are shifted and split.

**Fig. 5:**
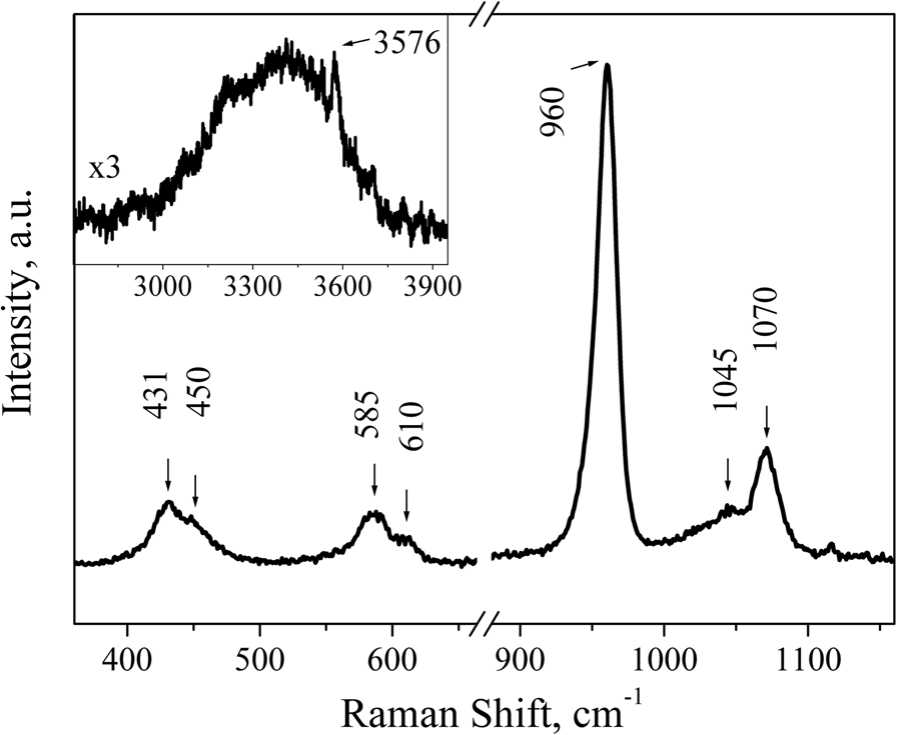
Raman spectrum of P2 powder

Figure 5 shows the Raman spectrum of P2 sample that is similar to the one of P1 sample. The main lines are observed in 360–1160 cm^−1^ spectral range. Source material demonstrates typical HAP vibrations. The phosphate *ν*_2_ vibrations (431 and 450 cm^−1^), phosphate *ν*_4_ vibrations (585 and 610 cm^−1^), and phosphate *ν*_1_ PO_4_^3−^ vibrations (960 cm^−1^) are seen. The structure observed in the 1020–1080-cm^−1^ range can be ascribed to the stretching *ν*_1_ mode of carbonate in B position, i.e., 1070 cm^−1^ [11–14], and the bending mode of carbonate at 1045 cm^−1^ [11] which overlaps with the wide background of the phosphate *ν*_3_ vibrations [14]. The broad band of low intensity in the range 3000–3750 cm^−1^ (see the inset in Fig. 5) can be attributed to the traces of water that is incorporated into the structure. A sharp peak at 3576 cm^−1^ is associated with the stretching vibration of the structural OH^−^group in HAP [15–17].

Raman spectra of the coatings have a number of differences from the spectra of source powders, namely, there is no band at 1070 cm^−1^ that indicates the escape of carbonate from B position of apatite lattice; the broad band 3000–3750 cm^−1^ disappears after coating deposition, indicating that water was removed from the crystal lattice. The similar carbonate and water escape during annealing of apatite was observed by other characterization methods [18, 19].

**Fig. 6:**
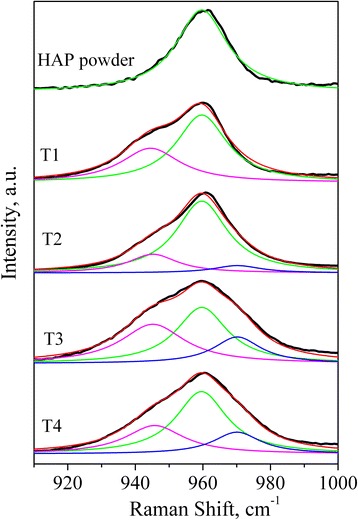
The experimental Raman spectra of initial HAP powder and coatings, their simulation, and fitting components: *black curves*—experimental Raman spectra of initial HAP powder and coatings; *red curves*—model spectra; *green curves*—fitting component at 960 cm^−1^; *magenta curves*—fitting component at 945 cm^−1^; *blue curves*—fitting component at 970 cm^−1^

The main differences in the Raman spectra of different coatings are the changes of position, intensity, and bandwidth of *ν*_*1*_ PO_4_^3−^ vibrations, which allows analyzing the composition and structural features of the coatings obtained. Fig. 6 shows the Raman features in the frequency range corresponding to *ν*_1_ for source HAP powder and coatings. It was found that *ν*_1_ component in both P1 and P2 powders is well fitted by single line at 960 cm^−1^. The best fitting for coatings was obtained using three fitting components with varied intensities but fixed positions and line widths. The first component at 960 cm^−1^ is caused by PO_4_^3−^ mode in HAP phase. To describe the low-frequency shoulder, the second fitting component at 945 cm^−1^ was used. Usually, the band at 945 cm^−1^ is assigned to amorphous calcium phosphate [20] that indicates of a highly disordered structure, although not necessarily a completely amorphous one. On the other hand, the position of this band corresponds to one of the *ν*_1_ components of PO_4_^3−^ vibrations of TTCP [21]. Since the X-ray diffraction patterns (Figs. 2 and 3) showed that the coatings have better crystallinity than the initial powders, therefore, the band at 945 cm^−1^ is more likely related to one of the *ν*_1_ components of PO_4_^3−^ vibrations of the TTCP phase. Moreover, the presence of TTCP phase was observed by XRD.

The third component is used to fit the high-frequency part of the spectrum. This component is placed at 970 cm^−1^ and can be assigned to *ν*_1_ PO_4_^3−^ of oxyapatite [22]. The presence of oxyapatite phase is clearly observed in Raman spectra, while XRD data do not allow distinguishing of HAP and oxyapatite phases. This finding correlates with the generally accepted fact that dehydrated HAP contains various intermediate phases, particularly oxyapatite [17]. As it is seen from Fig. 6, the more amorphous is the source powder, the larger is the content of oxyapatite in the ceramics, while the ratio between TTCP and HAP content is almost constant. The change of the distance to the substrate in the investigated range has less influence on the phase composition of the coating, although a slight increase in the amount of the oxyapatite phase is observed with increasing of the distance to the substrate.

### Electron Paramagnetic Resonance (EPR)

**Fig. 7:**
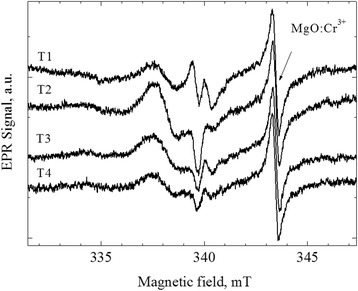
The experimental EPR spectra of coatings

EPR was used to investigate the local structure changes of HAP after the coating deposition. For the initial powders, the EPR signals were not observed. EPR spectra of the coatings are shown in Fig. 7. The difference in the EPR lineshape of coatings indicates that the EPR spectra have composite nature and are formed by contributions of at least two or three (as in the case of T1 sample) different types of paramagnetic centers.

**Fig. 8:**
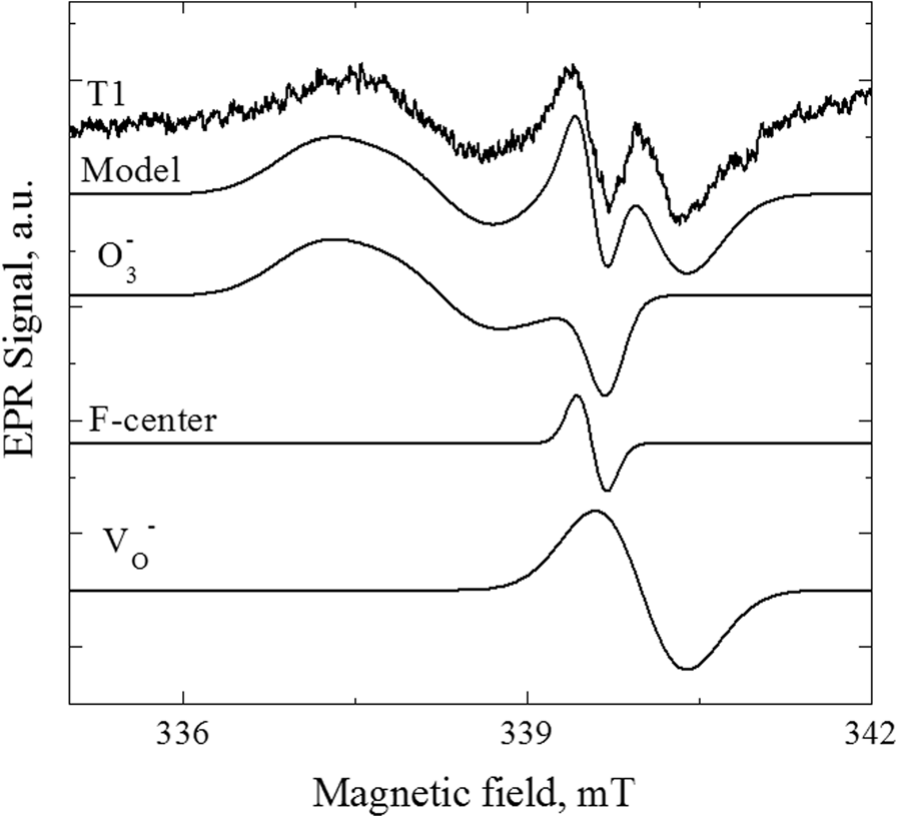
The experimental and modeled spectra of T1 sample, as well as the individual fitting components: *V*_*O*_^*-*^—electron localized on oxygen vacancy; *F-center*—electron localized on hydroxyl group vacancy; *О*_*3*_^*-*^—located between two vacant hydroxyl sites

Pretty well, description of the experimental EPR spectra of T2, T3, and T4 coatings can be achieved taking into account the contributions from two signals. The first fitting component is characterized by the parameters *g*_*x*_ = 2.0022, *g*_*y*_ = 2.0169, and *g*_*z*_ = 2.0108. These parameters are close to the values *g*_*x*_ = 2.0029, *g*_*y*_ = 2.0176, and *g*_*z*_ = 2.0105 observed for the center in HAP that is attributed to О_3_^-^ located between two vacant hydroxyl sites [23]. The small difference in the parameters indicates that it is the same О_3_^−^ center with slightly differed nearest surrounding. The second fitting component is the isotropic line with *g*_iso_ = 1.9990. The same signal was previously observed in modified HAP powders and attributed to V_O_^-^ center—an electron localized on an oxygen vacancy [24]. To describe the spectrum of T1 sample, one more line with *g*_iso_ = 2.0026 is needed. Isotropic lines in the range of 2.002–2.003 have been repeatedly observed in the annealed or mechanically treated HAP (*g* = 2.0019 (F-centers—electron localized on hydroxyl group vacancy) [25], *g* = 2.002 (unknown center) [26], *g* = 2.0029 (unknown center) [25], and *g* = 2.0032 (unknown center) [27]). The absence of hyperfine interaction features complicates the unambiguous identification of isotropic lines. Perhaps, the authors of all these works have dealt with the same center with slightly different nearest surrounding. In our opinion, the most suitable candidate for this center is the F-center. In Fig. 8, we show an example of modeling of the EPR spectrum of T1 coating by three fitting components described above.

### Mechanism of Paramagnetic Defects Formation

In a carbonate-containing HAP material, PO_4_^3-^ phosphate groups are partly replaced by carbonate CO_3_^2-^. It is obvious that the substitution CO_3_^2-^ → PO_4_^3-^ is not isovalent, and charge compensation is necessary for the existence of a stable structure. At present, the generally accepted mechanism for charge compensation at nonisovalent replacement CO_3_^2-^ → PO_4_^3-^ in HAP is the formation of vacancies of calcium and hydroxyl groups in neighboring lattice sites (see, e.g., [28]). The presence of V_OH_ in carbonated HAP was confirmed experimentally [29]. Carbonate replacement of B-type in the apatite structure leads to the formation of structural defects located on the sixfold axis (vacancy V_OH_ at OH site) and near this axis (vacancy V_Ca_ at Ca site); as a result, CO_3_^2−^-HAP becomes less stable as compared to stoichiometric HAP. Another source of hydroxyl group vacancies and additional structure changes is the influence of high temperatures. Loss of carbonate and water that are seen in the Raman spectra changes the lattice constant of HAP. Presumably, additional changes of the material structure occur when the heated HAP particles hit the substrate. This can lead to the displacement of atoms and redistribution of electrons. In particular, the charged paramagnetic defects are formed:

$$ \begin{array}{l}{\mathrm{V}}_{\mathrm{O}\mathrm{H}}+\mathrm{e}\to {{\mathrm{V}}_{\mathrm{O}\mathrm{H}}}^{-}\left(\mathrm{F}\hbox{-} \mathrm{center}\right);\hfill \\ {}{\mathrm{V}}_{\mathrm{O}}+\mathrm{e}\to {{\mathrm{V}}_{\mathrm{O}}}^{-}.\hfill \end{array} $$

At the same time, extended defects (nanopores) are formed along the sixfold axis; these defects can capture some molecules from the atmosphere, in particular, О_2_. This molecular О_2_ is most likely bonded to atomic oxygen (the residual of OH groups on the sixfold axis); as a result, the О_3_ structural unit is formed. Presumably, this process is similar to the A-type СО_3_^2-^-HAP formation (high-temperature annealing of HAP in СО_2_ flow) [30]:

$$ \begin{array}{l}2\mathrm{O}{\mathrm{H}}^{-}+\mathrm{C}{\mathrm{O}}_2\to \mathrm{C}{{\mathrm{O}}_3}^{2-}+{\mathrm{H}}_2\mathrm{O}\uparrow -\mathrm{A}\hbox{-} \mathrm{type}\ \mathrm{C}{{\mathrm{O}}_3}^{2-}\hbox{-} \mathrm{H}\mathrm{A}\mathrm{P}\;\mathrm{formation};\hfill \\ {}2\mathrm{O}{\mathrm{H}}^{-}+{\mathrm{O}}_2\to {{\mathrm{O}}_3}^{-}+{\mathrm{H}}_2\mathrm{O}\uparrow + \mathrm{e} - {{\mathrm{O}}_3}^{-}\mathrm{structural}\kern0.5em \mathrm{unit}\kern0.5em \mathrm{formation}.\hfill \end{array} $$

## Conclusions

Coatings obtained by detonation spraying of B-type carbonate-containing HAP are a mixture of two compounds: apatite and tetracalcium phosphate. The relative contributions of these phases depend on technological conditions of the coating production. The analysis of the Raman spectra demonstrated the appearance of oxyapatite phase in the ceramics caused by partial dehydration of HAP. The more amorphous is the initial powder, the more effective is the dehydrogenation. Transformations of the HAP structure occur predominantly along the sixfold axis and in its nearest surroundings. As a result, paramagnetic defects such as V_O_^-^, F-center, and О_3_^-^ center are formed. The above changes are caused by the sequential influence of various factors, the dominating one being the thermal heating of carbonate-containing HAP powder.

## Abbreviations

HAP: hydroxyapatite
SEM: scanning electron microscopy
TTCP: tetracalcium phosphate
XRD: X-ray diffraction
